# Impact of postoperative radiotherapy on combined local SCC events and survival in non-metastatic oral and pharyngeal squamous cell carcinoma

**DOI:** 10.1007/s12672-026-04449-8

**Published:** 2026-01-20

**Authors:** Yiquan Chen, Ruihuan Gao, Jingjing Wei, Xueying Liu, Yixuan Liang, Zhiyi Wu, Hai-jun Wu

**Affiliations:** 1 Xiangya Hospital, Central South University, Changsha, 410008 China; 2 School of Pharmacy, Jishou University, Jishou, 416000 China; 3 The Second Xiangya Hospital of Central South University, Changsha, 410011 China; 4 School of Integrated Chinese and Western Medicine, Hunan University of Chinese Medicine, Changsha, China; 5 Medical College, Central South University, Changsha, China

**Keywords:** Postoperative radiotherapy, Oral cavity cancer, Oropharyngeal cancer, Squamous cell carcinoma, SEER, Competing-risk analysis, Multiple primaries, Second primary tumors, Propensity score matching, Population-based study

## Abstract

**Background:**

Whether postoperative radiotherapy (PORT) reduces post-treatment malignant events in oral and pharyngeal squamous cell carcinoma (SCC) remains uncertain, particularly in population-based settings where true recurrence cannot be distinguished from second primary tumors (SPTs). Using SEER data (1975–2021), we evaluated the association between PORT and combined local SCC events—subsequent SCCs of identical morphology occurring within C00–C14—and assessed its impact on overall survival (OS).

**Methods:**

A retrospective cohort of 26,953 patients with first primary non-metastatic oral/pharyngeal SCC who underwent surgery was identified. PORT was defined as postoperative external-beam radiotherapy. The primary endpoint was combined local SCC events, reflecting SEER-captured recurrence- or SPT-like occurrences. Fine–Gray competing-risk models estimated subdistribution hazard ratios (sHRs), treating non-cancer death as the competing event. Poisson regression evaluated calendar-year trends. OS was assessed using Kaplan–Meier analysis with 1:1 propensity score matching (PSM) to adjust for baseline imbalances. Sensitivity analyses restricted to classical oral cavity SCC (C00–C06) and excluded all pharyngeal subsites.

**Results:**

Among 26,953 patients, 8,355 (31.0%) received PORT. PORT recipients had more adverse disease characteristics, including higher rates of regional-stage disease and high-grade morphology. During follow-up, 146 patients (0.54%) developed combined local SCC events, with lower incidence in the PORT group (0.7% vs. 0.2%, *P* < 0.001). PORT independently reduced the risk of combined events (sHR 0.34, 95% CI 0.20–0.59). Results were consistent across all subgroups and sensitivity analyses. After PSM, OS did not differ significantly between PORT and non-PORT groups (HR 0.97, 95% CI 0.89–1.12). Among patients who developed combined SCC events, OS likewise remained comparable.

**Conclusions:**

In this large, population-based study, PORT was associated with a substantially lower risk of registry-captured local SCC events across oral and pharyngeal subsites, although no adjusted survival advantage was observed. These findings underscore PORT’s role in improving local disease control while highlighting the need for recurrence-specific datasets to refine patient selection.This study provides real-world evidence on PORT effectiveness using a SEER-based composite endpoint tailored for registries lacking recurrence data.

**Supplementary Information:**

The online version contains supplementary material available at 10.1007/s12672-026-04449-8.

## Introduction

Globally, lip and oral cavity cancers account for around 377,000 new cases annually, with the majority of these tumors (> 90%) histologically diagnosed as squamous cell carcinoma [1–3]. “Tobacco use and alcohol consumption are well-established major risk factors for cancers of the oral cavity and pharyngeal subsites. Population-based registries (e.g., SEER) commonly group these anatomic sites under ‘oral cavity and pharynx cancers. [4–7] Surgical resection remains the standard-of-care for non-metastatic oral and pharyngeal squamous cell carcinoma, often followed by neck dissection [8–10]. Nevertheless, despite advances in surgical and reconstructive techniques, postoperative locoregional disease control remains a significant clinical challenge [11].

Postoperative radiotherapy (PORT) is widely used in head and neck oncology for patients with high-risk pathological features — including positive or close margins, extranodal extension (ENE), perineural or lymphovascular invasion, and nodal involvement — in order to improve locoregional control and reduce the risk of recurrence [12–15].However, its benefit in non-metastatic oral and pharyngeal SCC remains debated. Clinical practice varies substantially, partly due to heterogeneity in tumor biology across subsites, differences in surgical quality, and long-term toxicity concerns [16–19]. In addition, much of the existing literature focuses on selected subgroups—such as early oral cavity tumors or high-risk resected disease—leaving a gap in population-level evidence across the broader oral/pharyngeal spectrum [20–22].

A further challenge arises from the complexity of post-treatment events in head and neck cancer. True local recurrence, regional recurrence, and second primary tumors (SPTs) often share similar morphology and clinical behavior, and are difficult to distinguish reliably in large databases [23–27]. Importantly, the SEER registry does not contain a dedicated recurrence variable, and subsequent SCC diagnoses may represent either recurrence or SPTs [23]. Therefore, population-based studies must analyze combined post-treatment SCC events, recognizing that these reflect the overall burden of new malignant occurrences rather than pure recurrence.

Given these limitations and the lack of comprehensive real-world evidence, a large-scale population-based evaluation of PORT across non-metastatic oral and pharyngeal SCC is warranted. Using SEER data spanning 1975–2021, this study aims to (1) characterize the association between PORT and the risk of subsequent SCC events in oral and pharyngeal subsites; (2) compare overall survival between PORT-treated and untreated patients after adjusting for confounding using propensity score matching (PSM); and (3) explore temporal, age-related, and era-specific patterns of post-treatment SCC occurrence through dynamic risk analyses. Because SEER lacks recurrence-specific information, we define the primary endpoint as combined local events, encompassing both recurrence and SPTs.

## Methods

### Data source and study population

**Fig. 1:**
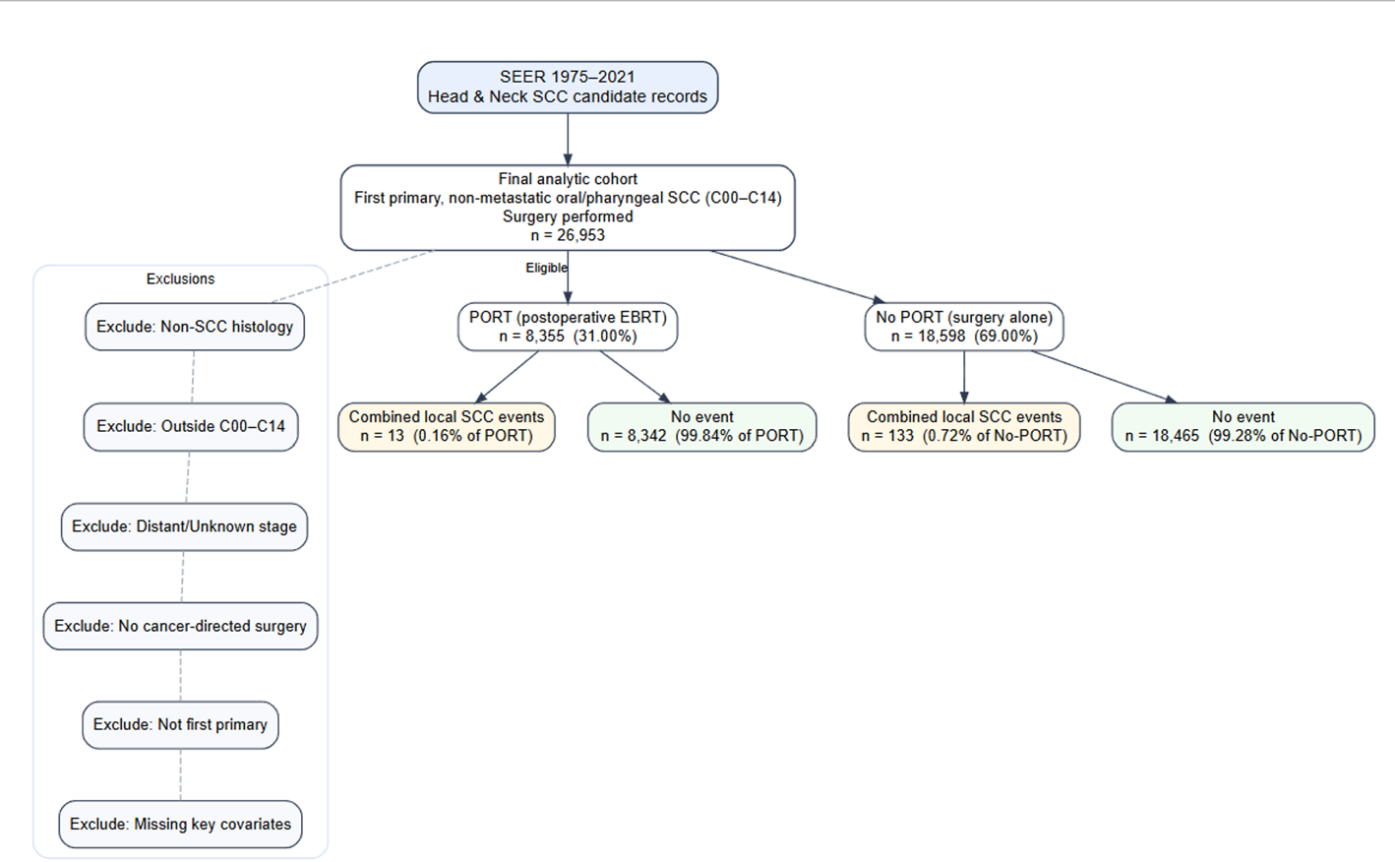
Study cohort selection flowchart

We conducted a retrospective population-based cohort study using data from the Surveillance, Epidemiology, and End Results (SEER) 17 registries (1975–2021). Eligible cases were first primary non-metastatic squamous cell carcinomas (SCC) of the oral cavity and pharyngeal subsites, identified using ICD-O-3 topography codes C00–C14. This classification is consistent with the SEER grouping of “oral cavity and pharynx cancers.”Because AJCC staging definitions have evolved substantially across the study period, SEER Historic Stage (Localized and Regional) was used to define a non-metastatic cohort. Cases with distant metastasis, non-SCC histology, missing surgery information, or unknown radiotherapy status were excluded.A sensitivity analysis restricted the cohort to classical oral cavity subsites (C00–C06) to assess whether inclusion of oropharyngeal and hypopharyngeal subsites materially influenced the results. See Fig. 1 for details.

### Treatment definition

Postoperative radiotherapy (PORT) was defined as external-beam radiotherapy administered after primary surgical resection, as recorded in SEER.Patients receiving brachytherapy, mixed modalities, or radiotherapy given before surgery were excluded to ensure treatment consistency.Chemotherapy status was extracted as a binary SEER variable (Yes/No).

### Outcome definition

SEER does not capture true recurrence, and subsequent malignancies are recorded only as new primary tumors. Therefore, consistent with prior registry-based studies, the primary endpoint was defined as: Combined local events = any subsequent SCC of identical morphology occurring in oral/pharyngeal subsites (C00–C14) after the index diagnosis.

This definition includes both: True local or regional recurrence, and Second primary tumors (SPTs) arising within the same anatomical region.Patients were followed from the date of initial cancer diagnosis to the occurrence of a combined local event, death, or end of follow-up (December 31, 2021). This endpoint is conservative and known to under-capture the full spectrum of clinical recurrences and second primaries; it was chosen to minimize misclassification in a registry that lacks a dedicated recurrence variable.

### Covariates

The following baseline variables were extracted from SEER and used in all adjusted models:

Age at diagnosis, Sex, Race (White / Black / Other), Year of diagnosis (continuous), Tumor, subsite (oral cavity / oropharynx / hypopharynx), Tumor size category, Histologic grade, SEER Historic Stage (Localized vs. Regional), Receipt of chemotherapy (Yes/No), PORT status (Yes/No).Variables unavailable in SEER—such as margin status, perineural invasion, lymphovascular invasion, and extracapsular extension—were acknowledged as unmeasured confounders.

### Statistical analysis

#### Baseline characteristics

Categorical variables were compared using chi-square tests and continuous variables using Student’s t-tests. Baseline characteristics before and after matching were summarized using standardized mean differences (SMDs).

### Competing-Risk analysis

Because deaths from causes other than oral/pharyngeal cancer may preclude observation of a post-treatment event, Fine–Gray subdistribution hazard models were used to estimate the association between PORT and combined local events.

#### Non-cancer death was treated as the competing event

Results were reported as subdistribution hazard ratios (sHRs) with 95% confidence intervals.

### Poisson regression

To evaluate time-dependent patterns of event occurrence across calendar years, multivariable Poisson regression models estimated adjusted rate ratios (aRRs), controlling for age and year of diagnosis. Poisson regression was used to evaluate temporal incidence patterns across calendar years, complementing the competing-risk analysis.

### Overall survival

Overall survival (OS) was analyzed using Kaplan–Meier curves and log-rank tests.Because PORT is often selectively offered to patients with high-risk pathological features not available in SEER, propensity score matching (PSM) was used to balance baseline covariates: Matching ratio: 1:1,Method: nearest neighbor, Caliper: 0.05 on the propensity score scale, Matching variables: age, year, race, subsite, tumor size, grade, Historic Stage, chemotherapy, Post-matching covariate balance was evaluated using SMD < 0.1 as acceptable.

### Dynamic risk analyses

To illustrate temporal and biological patterns of post-treatment SCC events, dynamic risk functions were generated stratified by latency interval (0–2, 2–5, > 5 years), age groups, and treatment era (1975–1990, 1991–2005, 2006–2021).

### Sensitivity analyses

Two prespecified sensitivity analyses were conducted: Restriction to classical oral cavity SCC (C00–C06),Exclusion of pharyngeal subsites (C09–C14),to evaluate the robustness of PORT associations when anatomical heterogeneity was reduced.

### Software

All analyses were performed using R version 4.4.2, including the packages survival, cmprsk, MatchIt, ggplot2, and dplyr.

A two-sided *P* < 0.05 was considered statistically significant.

Flow diagram showing inclusion and exclusion criteria applied to identify patients with first primary non-metastatic oral/pharyngeal squamous cell carcinoma (SCC) from SEER (1975–2021). The final analytic cohort included 26,953 eligible patients.

## Result

### Cohort characteristics

A total of 26,953 patients with first primary non-metastatic oral and pharyngeal squamous cell carcinoma (SCC) were identified from SEER (1975–2021). Among them, 8,355 (31.0%) received postoperative radiotherapy (PORT), and 18,598 (69.0%) underwent surgery alone. The median age was 63 years in the non-PORT group and 60 years in the PORT group, and tumor characteristics were substantially more adverse in PORT recipients, including higher rates of regional-stage disease (73.9% vs. 22.7%) and high-grade tumors (24.7% vs. 7.9%) (Table 1).

**Table 1:** Baseline characteristics of the study cohort by PORT status

	NRT	RT	*P*-value
	(*N* = 18598)	(*N* = 8355)	
Year.of.diagnosis			
Mean (SD)	1990 (12.0)	2000 (11.4)	< 0.001
Median [Min, Max]	1990 [1980, 2020]	2000 [1980, 2020]	
YearG			
> 2005	4696 (25.3%)	2546 (30.5%)	< 0.001
1975–1984	5090 (27.4%)	1491 (17.8%)	
1985–1994	4619 (24.8%)	2008 (24.0%)	
1995–2004	4193 (22.5%)	2310 (27.6%)	
Age			
Mean (SD)	62.3 (12.7)	60.1 (11.7)	< 0.001
Median [Min, Max]	63.0 [0, 84.0]	60.0 [14.0, 84.0]	
Missing	1136 (6.1%)	196 (2.3%)	
AgeG			
> 70	7185 (38.6%)	2216 (26.5%)	< 0.001
20–49	2510 (13.5%)	1272 (15.2%)	
50–70	8903 (47.9%)	4867 (58.3%)	
HistTumor			
Squamouscellcarcinoma	18,598 (100%)	8355 (100%)	< 0.001
GradeG			
Grade I/II	12,779 (68.7%)	5360 (64.2%)	< 0.001
Grade III/IV	1461 (7.9%)	2063 (24.7%)	
Unknown	4316 (23.2%)	861 (10.3%)	
Missing	42 (0.2%)	71 (0.8%)	
Race			
Black	468 (2.5%)	515 (6.2%)	< 0.001
Other (American Indian/AK Native, Asian/Pacific Islander)	934 (5.0%)	594 (7.1%)	
White	17,196 (92.5%)	7246 (86.7%)	
TumorsizeG			
< 2 cm	294 (1.6%)	35 (0.4%)	< 0.001
> 2 cm	11,185 (60.1%)	6230 (74.6%)	
Unknown	7119 (38.3%)	2090 (25.0%)	
Site			
Floor of Mouth	2665 (14.3%)	1614 (19.3%)	< 0.001
Gum and Oropharynx	3239 (17.4%)	2249 (26.9%)	
Lip	7156 (38.5%)	295 (3.5%)	
Tongue	5538 (29.8%)	4197 (50.2%)	
Chemotherapy			
No/Unknown	18,454 (99.2%)	6624 (79.3%)	< 0.001
Yes	144 (0.8%)	1731 (20.7%)	
Surgery			
Surgery performed	18,598 (100%)	8355 (100%)	< 0.001
Historic.stage			
Localized	14,381 (77.3%)	2177 (26.1%)	< 0.001
Regional	4217 (22.7%)	6178 (73.9%)	
local SCC events			
NO	18,465 (99.3%)	8342 (99.8%)	< 0.001
Yes	133 (0.7%)	13 (0.2%)	
Incubation period^a^			
Mean (SD)	93.7 (79.6)	74.1 (59.2)	0.505
Median [Min, Max]	71.0 [0, 361]	38.0 [15.0, 161]	
Missing	18,465 (99.3%)	8342 (99.8%)	
Survival.months			
Mean (SD)	131 (106)	87.4 (88.0)	< 0.001
Median [Min, Max]	107 [0, 562]	58.0 [0, 551]	

Comparisons reflect the distribution of demographic and tumor-related variables between patients who received postoperative radiotherapy (PORT) and those treated with surgery alone. Differences primarily reflect clinical selection for PORT based on adverse pathological suspicion not captured in SEER

PORT recipients were more likely to present with regional-stage disease, larger tumors, and higher-grade morphology (all *P* < 0.001), reflecting clinical selection for PORT in patients with potential adverse features that SEER does not record (e.g., margins, PNI, LVI, ENE). After propensity score matching, well-balanced 1:1 matched pairs were generated, with all standardized mean differences < 0.1.

**Fig. 2:**
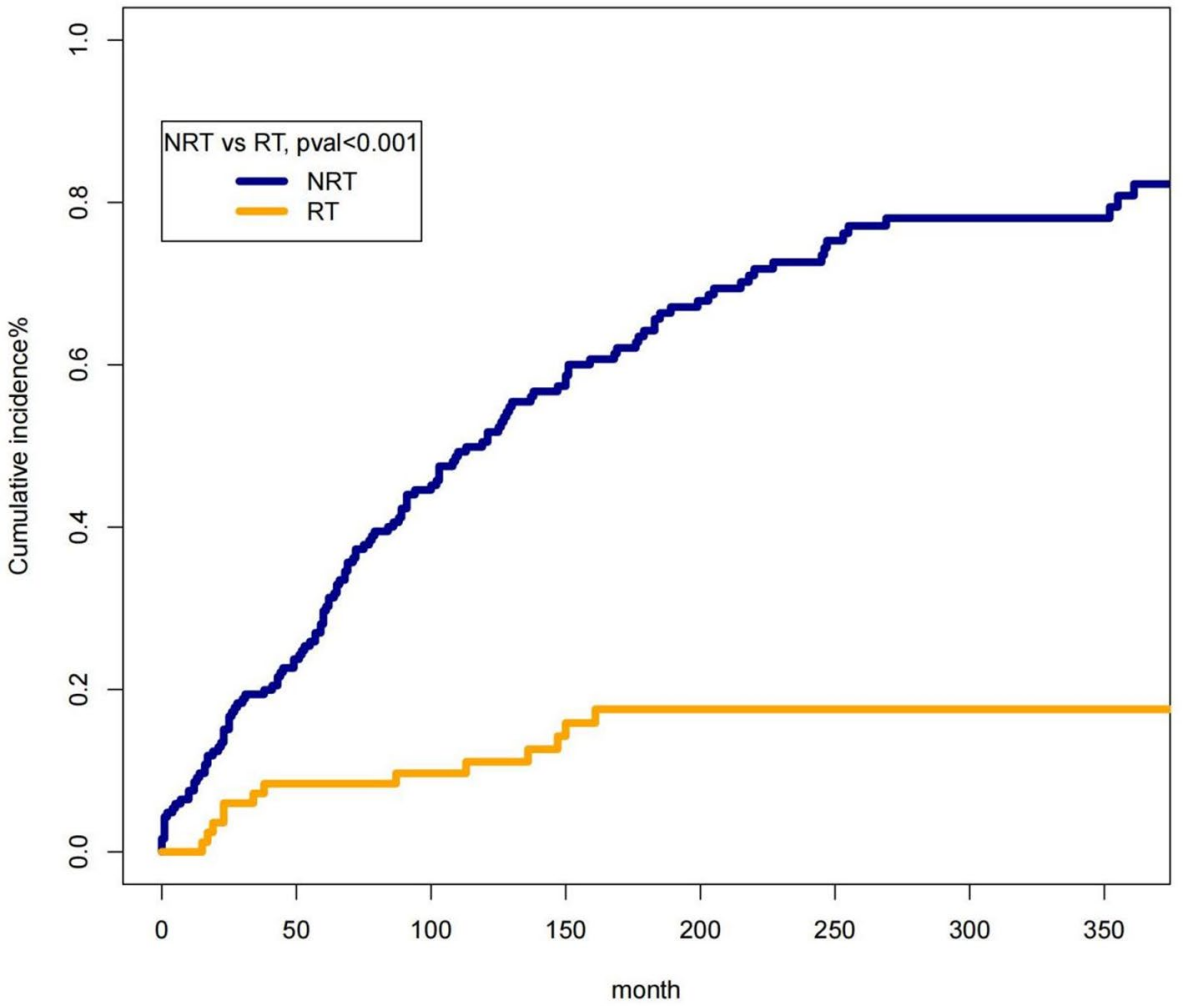
Temporal distribution of combined local SCC events

Cumulative incidence curves displaying registry-captured post-treatment SCC events over time. The early peak (0–24 months) likely reflects aggressive post-treatment events, while the later persistent incidence (≥ 5 years) is consistent with second primary tumors arising from field cancerization.

### Incidence and timing of Post-Treatment SCC events

Across the cohort, 146 patients (0.54%) developed a subsequent SCC within the oral or pharyngeal region during follow-up. These events represent registry-captured post-treatment SCC events, because SEER: does not record true recurrence, only logs new multiple primaries that satisfy SEER MP/H rules, and captures only those second SCCs that reappear within C00–C14 and share identical morphology.Thus, this endpoint reflects a conservative subset of clinically recognized recurrences and second primaries rather than the full clinical burden.The incidence differed between groups: PORT: The incidence was higher in the non-PORT group (0.7%) than in the PORT group (0.2%) (*P* < 0.001). Event timing showed a bimodal distribution (Fig. 2):0–24 months: sharp early peak, consistent with biologically plausible recurrence;60–120 months: persistent lower-level risk, consistent with field-cancerization–related second primaries;>10 years: steady low-level incidence.

**Fig. 3:**
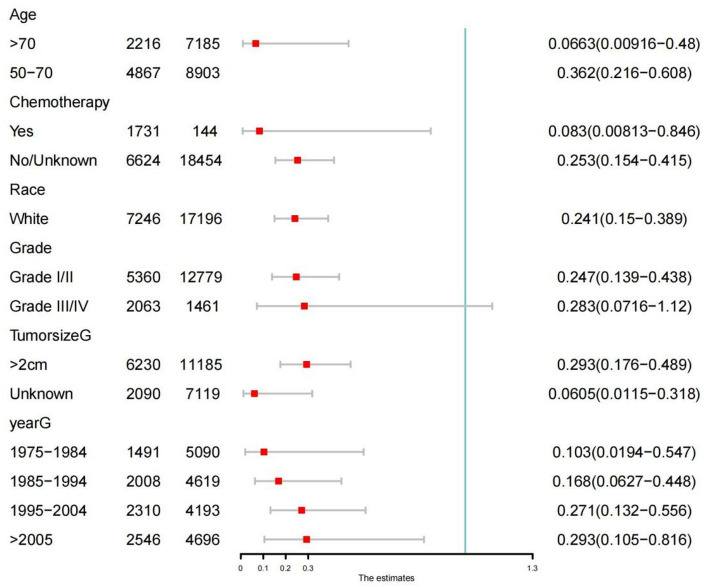
.Subgroup analyses of PORT and combined local SCC events

Subdistribution hazard ratios (Fine–Gray model) for PORT versus no PORT across age, sex, race, tumor subsite, tumor size category, histologic grade, and SEER Historic Stage. No significant interaction effects were observed.

### Effect of PORT on combined local SCC events

Using Fine–Gray competing-risk models with non-cancer death as the competing event, PORT was associated with a significantly lower risk of combined local SCC events:

Subdistribution HR (sHR): 0.34 (95% CI 0.20–0.59), *P* < 0.05 (eTable3).

Subgroup analyses showed consistent associations across: age (< 60 vs. ≥ 60 years), sex, race, tumor subsite (oral cavity, oropharynx, hypopharynx), tumor grade, tumor size category, SEER Historic Stage, with no significant interaction effects (Fig. 3).Two prespecified sensitivity analyses were performed: restricting to classical oral cavity subsites (C00–C06), and excluding all pharyngeal subsites, both yielding similar effect estimates, confirming robustness despite subsite heterogeneity.

**Fig. 4:**
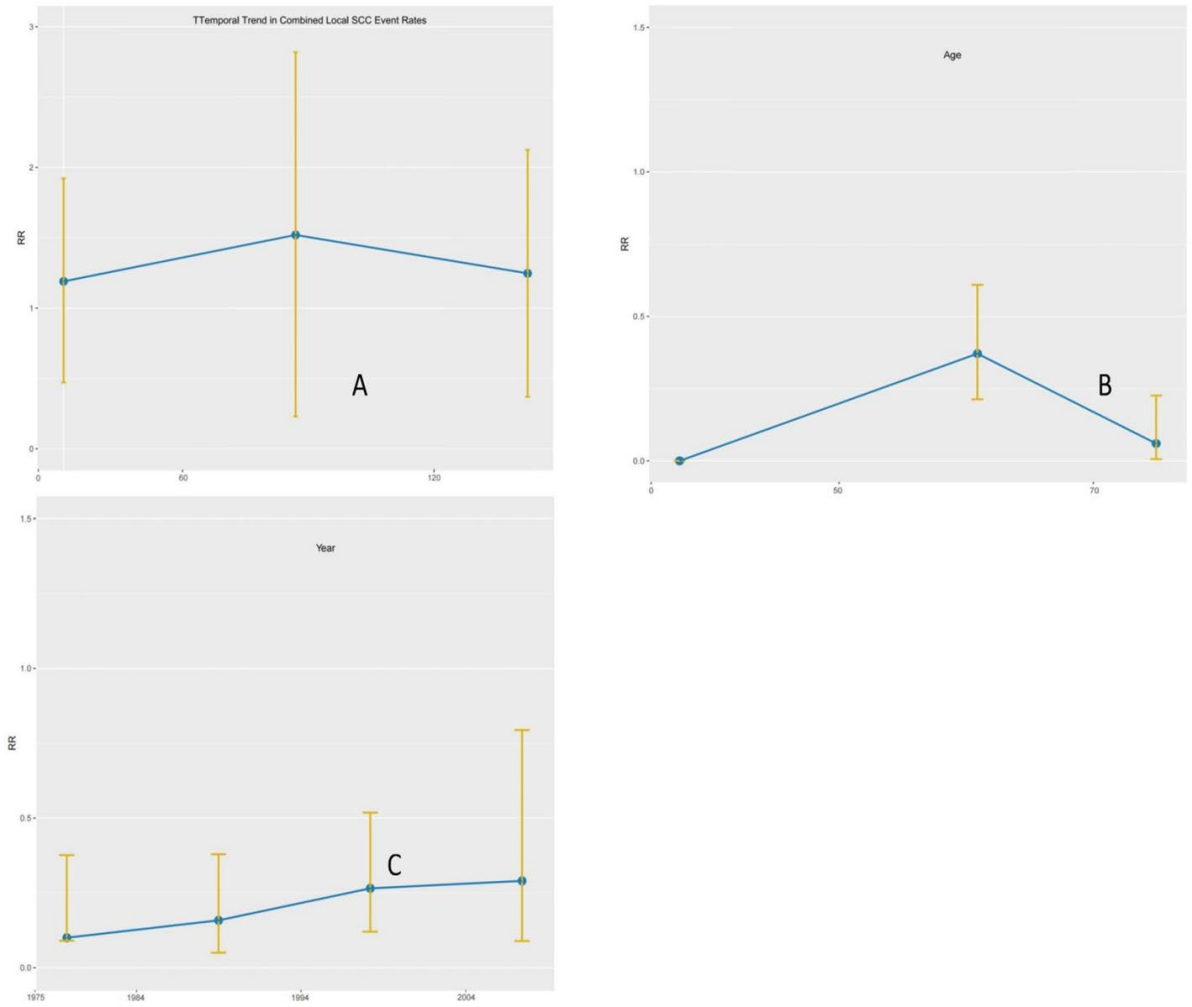
Dynamic event-risk patterns across age groups, diagnosis eras, and tumor subsites

Stratified temporal risk curves illustrating heterogeneity in early and late event patterns across clinical strata.

### Overall survival

In the unmatched cohort, PORT recipients exhibited poorer crude overall survival (log-rank *P* < 0.001), likely due to confounding by indication, whereby patients with unmeasured high-risk features were preferentially selected for PORT.

**Fig. 5:**
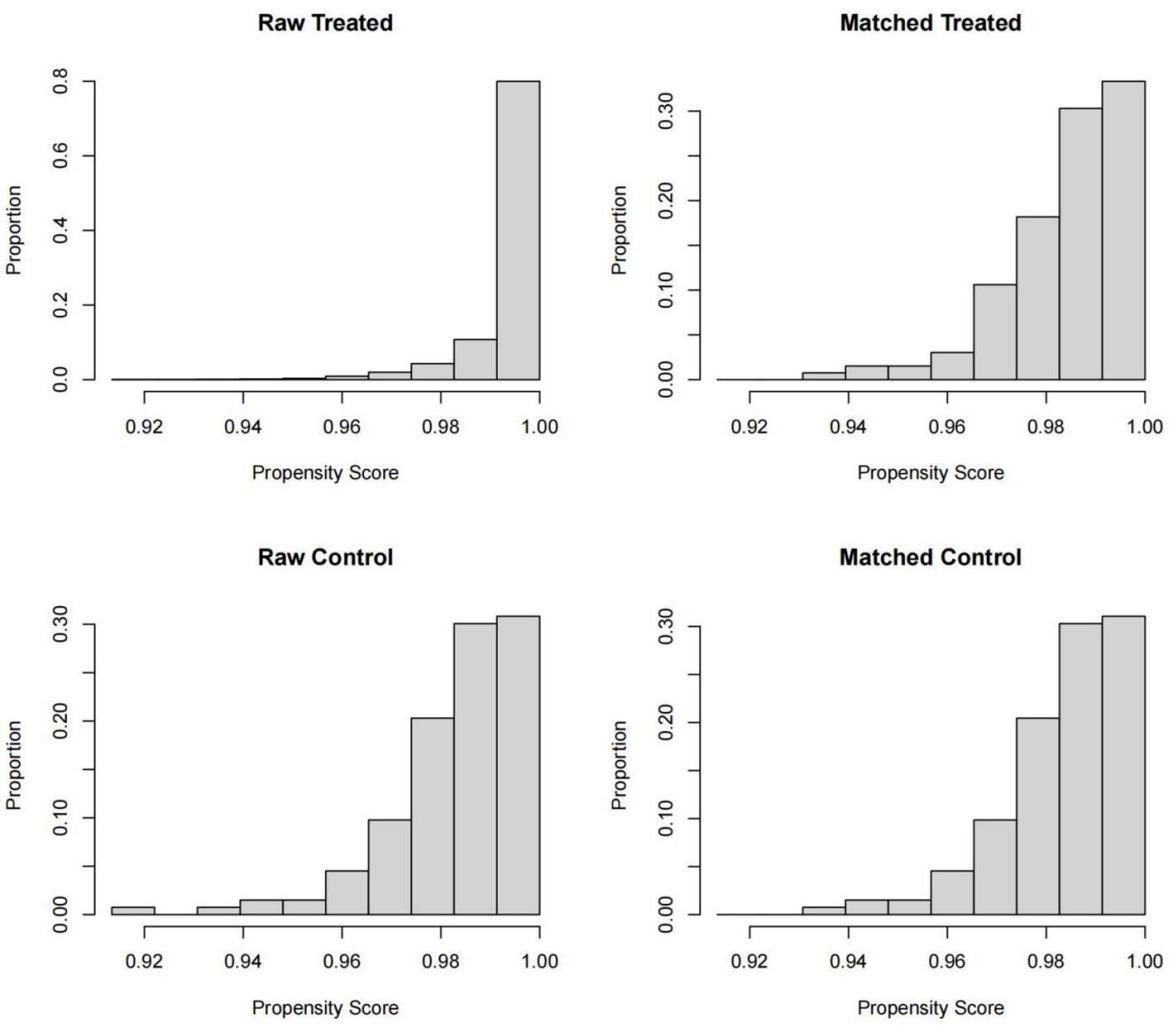
Propensity score distribution before and after matching

Kernel density plots showing the imbalance in propensity scores between PORT and non-PORT patients before matching and the marked improvement in covariate balance after 1:1 nearest-neighbor matching.

**Fig. 6:**
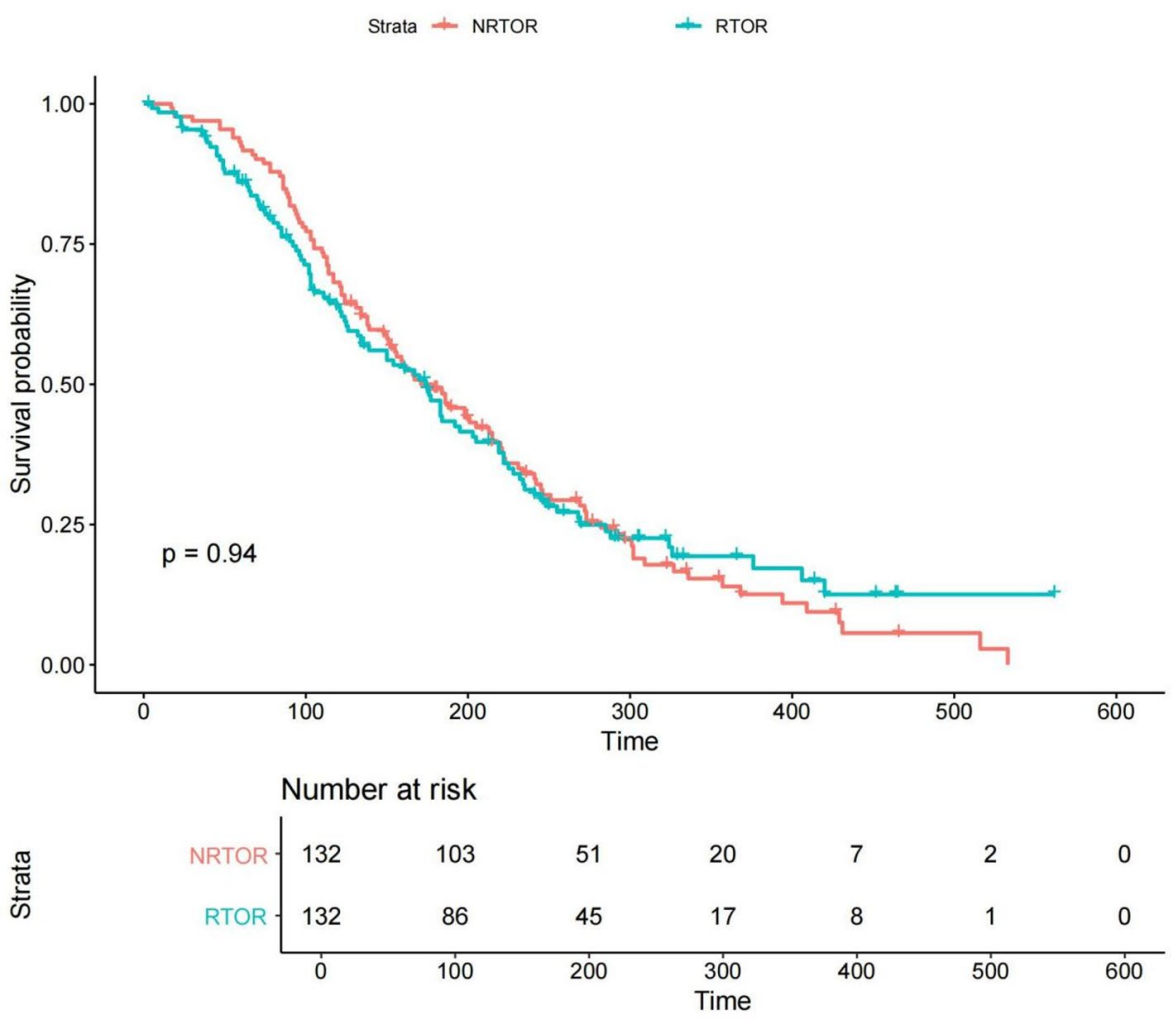
Overall survival among patients who developed post-treatment SCC events

Propensity score matching substantially improved covariate balance between the PORT and non-PORT groups (Fig. 5). After matching, overall survival became comparable (HR = 0.97, 95% CI 0.89–1.12; *P* = 0.46). Among patients who subsequently developed a combined local SCC event, survival likewise remained similar between treatment groups (Fig. 6).

Dynamic risk analyses further illustrated biologically meaningful heterogeneity:

Younger patients (< 60 years) exhibited a prolonged risk window beyond 5 years, consistent with cumulative risk for second primaries.Older patients (≥ 60 years) showed a concentrated early risk peak dominated by aggressive events.Patients diagnosed in the modern era (2006–2021) had the lowest overall event rates, reflecting advances in surgery, IMRT-based radiotherapy, and survivorship pathways. (Fig. 4)

Kaplan–Meier curves comparing overall survival in PORT versus non-PORT patients after experiencing a combined local SCC event. Survival differences were not statistically significant.

## Discussion

### Principal findings

In this population-based analysis of patients with non-metastatic oral and pharyngeal SCC, PORT was associated with a significantly lower risk of combined local SCC events, a conservative endpoint capturing subsequent SCCs recorded within C00–C14 under SEER multiple-primary rules. While this endpoint does not distinguish biologically between recurrence and second primary tumors (SPTs), the temporal pattern—an early peak followed by a later plateau—supports the clinical relevance of this combined measure. After adjustment using propensity score matching, PORT did not confer a statistically significant overall survival advantage, indicating that crude differences were largely driven by selection of higher-risk patients for PORT.

### Interpretation of Post-Treatment SCC events

A major methodological challenge in population datasets is the inability to differentiate true recurrence from SPTs, as SEER does not record recurrence. Our approach therefore prioritized specificity by defining post-treatment events strictly as subsequent SCCs of identical morphology recurring within oral or pharyngeal subsites. Although this captures fewer events than clinically observed, it minimizes misclassification and provides a reliable registry-level indicator of biologically meaningful post-treatment disease.

The strong early event peak likely reflects recurrence-like behavior, whereas the long-term risk aligns with the field-cancerization model, in which genetically altered mucosa predisposes to new primaries. This bimodal distribution supports the validity of our composite endpoint and underscores the need for nuanced interpretation in SEER-based studies.

### Effectiveness of PORT in contemporary and historical context

The association between PORT and reduced combined local SCC events aligns with its biological role in eradicating microscopic residual disease. Evidence for PORT primarily derives from high-risk head and neck SCC and smaller site-specific series; our study expands this by demonstrating a population-level benefit across a broader range of oral and pharyngeal subsites.

However, because SEER lacks critical pathological variables—margin status, perineural invasion, lymphovascular invasion, extranodal extension—the observed association cannot infer causality. Confounding by indication remains likely despite adjustment, as reflected by worse crude survival among PORT recipients.

### Subsite heterogeneity and sensitivity analyses

Reviewer 1 correctly highlighted the heterogeneity introduced by including oropharyngeal and soft-palate subsites. Although SEER classifies these under a shared oral/pharyngeal category and the subsites have overlapping etiologic exposures (tobacco, alcohol, chronic mucosal injury), biologic differences—particularly HPV involvement—are relevant. Our sensitivity analyses, which restricted to classical oral cavity SCC (C00–C06) and separately excluded all pharyngeal subsites, yielded results consistent with the primary model, demonstrating that the observed PORT effect is robust and not driven by subsite composition.

### Temporal trends and evolving standards of care

Event rates declined markedly across calendar decades. This trend is consistent with advancements in surgical technique, improvements in margin assessment, adoption of IMRT, multidisciplinary coordination, enhanced survivorship programs, and earlier detection in recent eras. These findings emphasize the necessity of interpreting long-term SEER analyses in the context of evolving treatment standards and highlight the limitations of single-era extrapolation.

### Overall survival and confounding by indication

The lack of a matched overall survival benefit from PORT likely reflects the complex interplay between competing mortality, unmeasured pathological factors, and patient selection. PORT is often reserved for individuals with clinically suspected high-risk features that are not captured in SEER, explaining the worse crude survival of the PORT group. Among patients who subsequently developed a combined local SCC event, survival trajectories were similar regardless of prior PORT, suggesting that once a new malignancy emerges, subsequent prognosis is primarily determined by tumor biology rather than historical treatment.

### Clinical implications

Our findings provide real-world evidence supporting the use of PORT to reduce post-treatment SCC events in appropriately selected patients. While survival differences were not evident after adjustment, the reduction in event burden reinforces the role of PORT in improving local disease control. The long-term risk pattern also highlights the importance of extended surveillance—particularly in younger patients and those with persistent field-cancerization risk factors.

### Limitations

Several limitations warrant consideration: (1) the inability of SEER to capture recurrence, margin status, PNI, LVI, ENE, HPV status, and treatment details; (2) the long study period encompassing substantial shifts in diagnostic and therapeutic practice; (3) potential residual confounding despite propensity matching; and(4) under-capture of SPTs occurring outside the oral/pharyngeal region.

These factors restrict causal inference and may underestimate the true clinical event burden.

## Conclusions

In this large population-based cohort, PORT was associated with a substantial reduction in combined local SCC events but no significant adjusted survival difference. The findings underscore the importance of PORT for local disease control and emphasize the need for modern, pathology-rich datasets to further clarify patient selection. Long-term surveillance remains essential given the dual risk of early aggressive events and later field-cancerization–related malignancies.

## Supplementary Information

Below is the link to the electronic supplementary material.

Supplementary Material 1.

## Data Availability

The datasets analyzed during the current study are publicly available from the Surveillance, Epidemiology, and End Results (SEER) Program of the National Cancer Institute ( https://seer.cancer.gov/ ). Access to SEER data requires submission of a research application. Processed data and supplementary tables/figures supporting the findings of this study are included in the manuscript and its supplementary information files.
