# Supplementary material for: Impact of postoperative radiotherapy on combined local SCC events and survival in non-metastatic oral and pharyngeal squamous cell carcinoma
Source: Discov Oncol. 2026 Jan 20;17:302. doi: 10.1007/s12672-026-04449-8 (PMC12905017; doi:10.1007/s12672-026-04449-8)
Supplement: Supplementary file 1 — Supplementary Material 1. [file 12672_2026_4449_MOESM1_ESM.docx]

**eTable1. SEER Historic Stage Definitions Used in This Study**

**Table title:**
SEER Historic Stage Definitions (1975–2021)

**Content：**

| **SEER Historic Stage** | **Definition (SEER)** | **Use in This Study** |
| --- | --- | --- |
| **Localized** | Cancer limited to organ of origin | Included as **non-metastatic** |
| **Regional** | Spread to regional lymph nodes / contiguous structures | Included as **non-metastatic** |
| **Distant** | Metastatic disease | Excluded |
| **Unknown** | Staging cannot be determined | Excluded |

**Footnote:**
Historic Stage was used instead of AJCC due to staging system changes (1975–2021). Only localized and regional stages were included.The description of tumor stage of cancer in SEER Program was derived from SEER official website (<https://seer.cancer.gov/>)

**eTable2. Baseline Characteristics by Combined Local SCC Events (RT vs NRT)**

|  |  |  |  |
| --- | --- | --- | --- |
|  | **NRT** | **RT** | **P-value** |
|  | **(N=133)** | **(N=13)** |  |
| **Chemotherapy** |  |  |  |
| No/Unknown | 132 (99.2%) | 12 (92.3%) | <0.001 |
| Yes | 1 (0.8%) | 1 (7.7%) |  |
| **Stage** |  |  |  |
| Localized | 116 (87.2%) | 6 (46.2%) | <0.001 |
| Regional | 17 (12.8%) | 7 (53.8%) |  |
| **Survival.months** |  |  |  |
| Mean (SD) | 188 (109) | 148 (83.2) | <0.001 |
| Median [Min, Max] | 160 [17.0, 533] | 144 [35.0, 279] |  |
| **Age.recode.with.single.ages.and.90.** |  |  |  |
| Mean (SD) | 62.2 (12.8) | 57.2 (7.82) | <0.001 |
| Median [Min, Max] | 64.0 [22.0, 84.0] | 55.0 [49.0, 73.0] |  |
| Missing | 1 (0.8%) | 0 (0%) |  |
| **AgeG** |  |  |  |
| >70 | 49 (36.8%) | 1 (7.7%) | <0.001 |
| 20-49 | 22 (16.5%) | 0 (0%) |  |
| 50-70 | 62 (46.6%) | 12 (92.3%) |  |
| **Reason.no.cancer.directed.surgery** |  |  |  |
| Surgery performed | 133 (100%) | 13 (100%) | <0.001 |
| **Site.recode.ICD.O.3.WHO.2008** |  |  |  |
| Floor of Mouth | 8 (6.0%) | 2 (15.4%) | <0.001 |
| Gum and Oropharynx | 13 (9.8%) | 4 (30.8%) |  |
| Lip | 106 (79.7%) | 1 (7.7%) |  |
| Tongue | 6 (4.5%) | 6 (46.2%) |  |
| **After time** **(months)** |  |  |  |
| Mean (SD) | 93.7 (79.6) | 74.1 (59.2) | 0.505 |
| Median [Min, Max] | 71.0 [0, 361] | 38.0 [15.0, 161] |  |
| **Year.of.diagnosis** |  |  |  |
| Mean (SD) | 1990 (10.5) | 2000 (10.6) | <0.001 |
| Median [Min, Max] | 1990 [1980, 2010] | 2000 [1980, 2020] |  |
| **yearG** |  |  |  |
| >2005 | 19 (14.3%) | 3 (23.1%) | <0.001 |
| 1975-1984 | 33 (24.8%) | 1 (7.7%) |  |
| 1985-1994 | 41 (30.8%) | 3 (23.1%) |  |
| 1995-2004 | 40 (30.1%) | 6 (46.2%) |  |
| **GradeG** |  |  |  |
| Grade I/II | 88 (66.2%) | 9 (69.2%) | <0.001 |
| Grade III/IV | 5 (3.8%) | 2 (15.4%) |  |
| Unknown | 40 (30.1%) | 2 (15.4%) |  |
| **HistTumor** |  |  |  |
| Squamouscellcarcinoma | 133 (100%) | 13 (100%) | <0.001 |
|  |  |  |  |

**eTable3. Fine–Gray and Poisson Regression for Combined Local SCC Events**

| Characteristic | Univariable Competing Risk Regression | | Multivariable Competing Risk Regression | | Poisson regression (RT vs NRT) | |
| --- | --- | --- | --- | --- | --- | --- |
|  | HR (95% Cl) | P | HR (95% Cl) | P | Adjusted RR (95% CI) | P |
| Age at diagnosis, per year | 1(0.99-1.01) | 0.91 | - | - | - | - |
| Year of diagnosis, per year | 0.991(0.98-1) | 0.16 | - | - | - | - |
| Race |  |  |  |  |  |  |
| White | 1 |  | 1 |  | - |  |
| Other | 0.211(0.0808-0.551) | <0.05 | 0.25(0.0954-0.654) | <0.05 | - | - |
| Tumor size (cm) |  |  |  |  |  |  |
| <2 | 1 |  | - |  | - |  |
| ≥2 | 0.589(0.226-1.54) | 0.36 | - | - | - | - |
| Chemotherapy |  |  |  |  |  |  |
| No | 1 |  | 1 |  | - |  |
| Yes | 0.2(0.0619-0.643) | <0.05 | 0.534(0.1530-1.861) | 0.58 | - | - |
| Stage |  |  |  |  |  |  |
| Localized | 1 |  | 1 |  | - |  |
| Regional | 0.316(0.219-0.457) | <0.05 | 0.502(0.3375-0.747) | <0.05 | - | - |
| Radiation therapy |  |  |  |  |  |  |
| No | 1 |  | 1 |  | 1 |  |
| Yes | 0.221(0.137-0.356) | <0.05 | 0.343(0.2007-0.587) | <0.05 | 0.215(0.129-0.339) | <0.05 |

Fine–Gray model treated non-cancer death as a competing event. Poisson regression evaluated temporal incidence patterns adjusted for age and calendar year.

**eTable4. Subgroup Analyses (Fine–Gray Competing-Risk Model)**

| Subgroup | RT | NRT | HR(95%) | P |
| --- | --- | --- | --- | --- |
| Age |  |  |  |  |
| >70 | 2216 | 7185 | 0.0663(0.00916-0.48) | <0.05 |
| 50-70 | 4867 | 8903 | 0.362(0.216-0.608) | <0.05 |
| Chemotherapy |  |  |  |  |
| Yes | 1731 | 144 | 0.083(0.00813-0.846) | <0.05 |
| No/Unknown | 6624 | 18454 | 0.253(0.154-0.415) | <0.05 |
| Race |  |  |  |  |
| White | 7246 | 17196 | 0.241(0.15-0.389) | <0.05 |
| Grade |  |  |  |  |
| Grade I/II | 5360 | 12779 | 0.247(0.139-0.438) | <0.05 |
| Grade III/IV | 2063 | 1461 | 0.283(0.0716-1.12) | - |
| TumorsizeG |  |  |  |  |
| >2cm | 6230 | 11185 | 0.293(0.176-0.489) | <0.05 |
| Unknown | 2090 | 7119 | 0.0605(0.0115-0.318) |  |
| YearG |  |  |  |  |
| 1975-1984 | 1491 | 5090 | 0.103(0.0194-0.547) | <0.05 |
| 1985-1994 | 2008 | 4619 | 0.168(0.0627-0.448) | <0.05 |
| 1995-2004 | 2310 | 4193 | 0.271(0.132-0.556) | <0.05 |
| >2005 | 2546 | 4696 | 0.293(0.105-0.816) | <0.05 |
| Stage |  |  |  |  |
| Localized | 2177 | 14381 | 0.341(0.171-0.678) | <0.05 |
| Regional | 6178 | 4217 | 0.285(0.136-0.596) | <0.05 |

**eTable5.Baseline Characteristics After Propensity Score Matching**

|  | **NRT** | **RT** | **TRUE** | P |
| --- | --- | --- | --- | --- |
|  | **(N=132)** | **(N=132)** | **(N=264)** |  |
| **Survival.months** |  |  |  | 0.34 |
| Mean (SD) | 187 (108) | 166 (115) | 177 (112) |  |
| Median [Min, Max] | 160 [17.0, 533] | 135 [3.00, 562] | 153 [3.00, 562] |  |
| **ageG** |  |  |  | 0.07 |
| >70 | 49 (37.1%) | 66 (50.0%) | 115 (43.6%) |  |
| 20-49 | 22 (16.7%) | 22 (16.7%) | 44 (16.7%) |  |
| 50-70 | 61 (46.2%) | 44 (33.3%) | 105 (39.8%) |  |
| **TumorsizeG** |  |  |  | 0.35 |
| <2cm | 2 (1.5%) | 0 (0%) | 2 (0.8%) |  |
| >2cm | 74 (56.1%) | 73 (55.3%) | 147 (55.7%) |  |
| Unknown | 56 (42.4%) | 59 (44.7%) | 115 (43.6%) |  |
| **Chemotherapy.recode..yes..no.unk.** |  |  |  | 1 |
| No/Unknown | 131 (99.2%) | 131 (99.2%) | 262 (99.2%) |  |
| Yes | 1 (0.8%) | 1 (0.8%) | 2 (0.8%) |  |
| **Sex** |  |  |  | 1 |
| Male | 132 (100%) | 131 (99.2%) | 263 (99.6%) |  |
| Female | 0 (0%) | 1 (0.8%) | 1 (0.4%) |  |
| **Race.recode..White..Black..Other.** |  |  |  | 0.6 |
| Other (American Indian/AK Native, Asian/Pacific Islander) | 3 (2.3%) | 3 (2.3%) | 6 (2.3%) |  |
| White | 129 (97.7%) | 128 (97.0%) | 257 (97.3%) |  |
| Black | 0 (0%) | 1 (0.8%) | 1 (0.4%) |  |
| **yearG** |  |  |  | 0.08 |
| >2005 | 19 (14.4%) | 24 (18.2%) | 43 (16.3%) |  |
| 1975-1984 | 33 (25.0%) | 24 (18.2%) | 57 (21.6%) |  |
| 1985-1994 | 40 (30.3%) | 56 (42.4%) | 96 (36.4%) |  |
| 1995-2004 | 40 (30.3%) | 28 (21.2%) | 68 (25.8%) |  |
